# Supplementary material for: A Critical View of the Application of the APEX Software (Aqueous Photochemistry of Environmentally-Occurring Xenobiotics) to Predict Photoreaction Kinetics in Surface Freshwaters
Source: Molecules. 2019 Dec 18;25(1):9. doi: 10.3390/molecules25010009 (PMC7017383; doi:10.3390/molecules25010009)
Supplement: Supplementary file 1 [file molecules-25-00009-s001.zip › 3.2.4_gcc-4.4.0.zip!/3.2.4_gcc-4.4.0/3.2.4_gcc-4.4.0/doc/octave/html/interpreter/Evaluating-Polynomials.html]

Evaluating Polynomials - Untitled


Next: Finding Roots,
Up: Polynomial Manipulations


---

### 27.1 Evaluating Polynomials

The value of a polynomial represented by the vector c can be evaluated
at the point x very easily, as the following example shows:

```
     N = length(c)-1;
     val = dot( x.^(N:-1:0), c );
```

While the above example shows how easy it is to compute the value of a
polynomial, it isn't the most stable algorithm. With larger polynomials
you should use more elegant algorithms, such as Horner's Method, which
is exactly what the Octave function `polyval` does.

In the case where x is a square matrix, the polynomial given by
c is still well-defined. As when x is a scalar the obvious
implementation is easily expressed in Octave, but also in this case
more elegant algorithms perform better. The `polyvalm` function
provides such an algorithm.

— Function File: y = **polyval** (p, x)  
— Function File: y = **polyval** (p, x, [], mu)  
> Evaluate the polynomial at of the specified values for x. When mu
> is present evaluate the polynomial for (x-mu(1))/mu(2).
> If x is a vector or matrix, the polynomial is evaluated for each of
> the elements of x.
> — Function File: [y, dy] = **polyval** (p, x, s)  
> — Function File: [y, dy] = **polyval** (p, x, s, mu)  
> > In addition to evaluating the polynomial, the second output
> > represents the prediction interval, y +/- dy, which
> > contains at least 50% of the future predictions. To calculate the
> > prediction interval, the structured variable s, originating
> > form `polyfit', must be present.
> >
> >
> >
> > **See also:** polyfit, polyvalm, poly, roots, conv, deconv, residue, filter, polyderiv, polyinteg.

— Function File: **polyvalm** (c, x)  
> Evaluate a polynomial in the matrix sense.
>
> `polyvalm (`c`,` x`)` will evaluate the polynomial in the
> matrix sense, i.e., matrix multiplication is used instead of element by
> element multiplication as is used in polyval.
>
> The argument x must be a square matrix.
>
>
>
> **See also:** polyval, poly, roots, conv, deconv, residue, filter, polyderiv, polyinteg.
